# CRISPR–Cas-Mediated Reprogramming Strategies to Overcome Antimicrobial Resistance

**DOI:** 10.3390/pharmaceutics18010095

**Published:** 2026-01-11

**Authors:** Byeol Yoon, Jang Ah Kim, Yoo Kyung Kang

**Affiliations:** 1 Research Institute of Pharmaceutical Sciences, College of Pharmacy, Gyeongsang National University, Jinju 52828, Republic of Korea; 2 Hamlyn Centre for Robotic Surgery, Department of Mechanical Engineering, Imperial College London, London SW7 2AZ, UK; j.a.kim@imperial.ac.uk

**Keywords:** CRISPR–Cas, antimicrobial resistance (AMR), superbug transmission control, horizontal gene transfer (HGT), precision antimicrobials

## Abstract

Antimicrobial resistance (AMR) is escalating worldwide, posing a serious threat to global public health by driving infections that are no longer treatable with conventional antibiotics. CRISPR–Cas technology offers a programmable and highly specific therapeutic alternative by directly targeting the genetic determinants responsible for resistance. Various CRISPR systems can restore antibiotic susceptibility and induce selective bactericidal effects by eliminating resistance genes, disrupting biofilm formation, and inhibiting virulence pathways. Moreover, CRISPR can suppress horizontal gene transfer (HGT) by removing mobile genetic elements such as plasmids, thereby limiting the ecological spread of AMR across humans, animals, and the environment. Advances in delivery platforms—including conjugative plasmids, phagemids, and nanoparticle-based carriers—are expanding the translational potential of CRISPR-based antimicrobial strategies. Concurrent progress in Cas protein engineering, spatiotemporal activity regulation, and AI-driven optimization is expected to overcome current technical barriers. Collectively, these developments position CRISPR-based antimicrobials as next-generation precision therapeutics capable of treating refractory bacterial infections while simultaneously suppressing the dissemination of antibiotic resistance.

## 1. Introduction

Antimicrobial resistance (AMR) has emerged as one of the most serious global public health challenges, threatening to undermine the effectiveness of modern medicine and making even common bacterial infections increasingly difficult to treat. In 2019, AMR was directly responsible for approximately 1.27 million deaths and associated with nearly 5 million deaths worldwide [1]. A subsequent long-term analysis updated these figures to 1.14 million direct deaths and 4.71 million associated deaths in 2021, confirming that the burden of AMR remained substantial even during the COVID-19 pandemic [2]. The World Health Organization (WHO) has designated AMR as a major global health threat, warning that failure to address the current gaps in antibiotic innovation and access could cost the global economy up to USD 100 trillion by 2050 [3]. This projection implies that even common infections such as pneumonia, tuberculosis, gonorrhea, and salmonellosis could become untreatable, ultimately destabilizing the foundations of modern medicine, including surgery, cancer therapy, and critical care.

The primary drivers of AMR include inappropriate antibiotic prescriptions, overuse in agriculture and livestock, inadequate dosing, and insufficient infection control systems, all of which generate strong selective pressures that facilitate the emergence and spread of resistant strains [4]. These problems are further exacerbated by the distribution of counterfeit or substandard medicines, the release of antibiotic residues into the environment, and the horizontal transfer of resistance genes. More fundamentally, the discovery of new classes of low-molecular-weight antimicrobial agents has been virtually stagnant for decades. Most new antibiotics reported in recent years are merely structural derivatives of existing scaffolds, lacking any genuinely novel mechanism of action [5]. This widening gap between the rapid evolution of bacterial resistance and the stagnation of antibiotic innovation underscores the urgent need for alternative antimicrobial strategies.

Several promising approaches have been explored as potential alternatives to conventional antibiotics, including bacteriophage therapy, antimicrobial peptides, vaccines, and microbiome-based interventions. However, these strategies face significant limitations, such as narrow host range, stability issues, immunogenicity, and inconsistent efficacy in clinical trials [6,7,8]. Against this backdrop, clustered regularly interspaced short palindromic repeats (CRISPR) and CRISPR-associated (Cas) proteins have emerged as versatile and programmable molecular tools. Originally discovered as adaptive immune systems in bacteria and archaea, CRISPR–Cas systems have since been repurposed as targeted nucleic acid remodeling platforms capable of precise DNA or RNA cleavage and editing [9,10,11,12,13].

Unlike existing antibiotics, which non-specifically interfere with essential bacterial processes, CRISPR technology offers exceptional programmability and sequence specificity, enabling the selective targeting of genes located on plasmids or chromosomes to control AMR [14,15]. Beyond simple gene editing, CRISPR systems can regulate transcription, modulate the epigenome, and even manipulate microbial community dynamics [16,17]. These multifunctional capabilities present the potential to restore antibiotic sensitivity in resistant strains, suppress horizontal gene transfer (HGT), and minimize collateral impacts on commensal microbiota [18,19,20]. To date, CRISPR–Cas–based antimicrobial strategies have been most extensively investigated in clinically significant, high-burden bacterial pathogens—including *Escherichia coli*, *Klebsiella pneumoniae*, *Staphylococcus aureus*, and other multidrug-resistant Gram-negative and Gram-positive species—thereby underscoring their strong translational relevance for antimicrobial resistance control. Collectively, CRISPR-based antimicrobials represent a paradigm shift toward narrow-spectrum, precision-targeted strategies capable of addressing the growing global threat of AMR.

## 2. CRISPR–Cas Systems: Mechanism of Action and Diversity

The CRISPR–Cas system is an adaptive immune defense mechanism in prokaryotes that protects cells by recognizing and cleaving foreign nucleic acids [9]. This process occurs in three main stages. First, during the adaptation stage, foreign DNA fragments are inserted into the CRISPR array as spacers. Next, during the processing stage, the array is transcribed and processed to form mature crRNA. This crRNA guides the Cas protein to cleave complementary target nucleic acids, a process termed the interference stage [10]. This mechanism induces rapid defense against reinfection by the same pathogen and also functions as a crucial barrier against the spread of horizontal gene transfer, such as plasmids carrying antibiotic resistance genes [12].

Structurally, CRISPR–Cas systems are classified into Class 1 and Class 2, with Class 1 utilizing a multi-protein complex and Class 2 employing a single large protein as the effector [16]. Consequently, the primary focus of research and applications has converged on the Class 2 CRISPR-Cas family, which utilizes a single protein based on the high efficiency of the single effector. Representative examples include Cas9 (Type II), Cas12 (Type V), and Cas13 (Type VI), which perform DNA double-strand cleavage, PAM-dependent staggered double-strand breaks (DSBs), and RNA targeting, respectively. These provide the foundation not only for genome editing and transcriptional regulation but also for developing molecular diagnostic platforms [11,16,17,21].

However, the clinical application of CRISPR–Cas still faces significant constraints, including target limitations due to PAM dependency, off-target cuts, immune responses, and reduced delivery efficiency [22]. Notably, to improve delivery efficiency, compact orthologs such as SaCas9, CjCas9, and NmCas9 have been identified, enhancing the suitability of AAV vectors constrained by packaging capacity [23,24]. Furthermore, enzyme size optimization is a key challenge for clinical translation. Ultra-small enzymes (≤1,000 aa) like CasΦ, Cas12f (CasX), and Cas14 are gaining attention as candidates that expand therapeutic delivery possibilities [25,26,27]. Such ultra-small structures are suitable not only for AAV but also for various non-viral delivery strategies like LNP and polymeric carriers [28,29]. Issues of reduced activity due to size reduction can be mitigated through rational design approaches like domain deletion or module exchange, combined with iterative evolutionary techniques. In addition, through protein engineering, variants that mitigate PAM constraints, such as xCas9, SpCas9-NG, and SpRY [30,31], and high-fidelity variants like eSpCas9, SpCas9-HF1, and HypaCas9 [32,33,34] have been developed. Moreover, cases have been reported where continuous evolution techniques like PACE (phage-assisted continuous evolution) were applied to simultaneously improve accuracy and stability while enhancing gene editing efficiency [35,36,37].

In terms of functional expansion, dCas9, which lacks DSB cleavage activity, is applied to CRISPRi/a and epigenetic regulation [38], while nCas9, possessing only single-strand cleavage activity, reduces cellular toxicity while improving homology-directed repair (HDR) efficiency [39]. Furthermore, fusing Cas proteins with Base editors or Prime editors enables efficient specific base substitutions or small-scale insertion/deletion corrections [40,41]. In addition, spatiotemporal control based on light-inducible, ligand-dependent, or split-Cas9 systems can induce CRISPR activity only at the infection site or at specific time points. This effectively reduces unnecessary off-target cuts, unintended gene disruption in non-target tissues, and unexpected side effects that may occur during sensitive stages of cell differentiation or tissue development [42,43,44]. The structural features and applications of these representative Cas enzymes are summarized in Table 1.

## 3. DNA Repair Pathways Influencing CRISPR–Cas-Mediated Genome Editing in Bacteria

The success of gene editing in bacteria using the CRISPR–Cas system depends not only on the cleavage activity of Cas proteins but also on the cellular DNA repair pathways that process the resulting DSBs [45,46,47,48]. Bacteria generally repair DSBs via two main mechanisms—homologous recombination (HR) and non-homologous end joining (NHEJ)—analogous to those in mammalian cells [45,47]. In certain strains, alternative end joining (A-EJ or MMEJ) can also contribute [45,48].

Most bacteria primarily employ HR mediated by RecA together with RecBCD (or AddAB/AdnAB, particularly in *Mycobacteria*) and RecFOR pathways [45]. For instance, *Escherichia coli* (*E. coli*), *Helicobacter pylori*, *Haemophilus influenzae*, *Lactococcus lactis*, and others efficiently perform precise DNA repair through HR, often coupling recombination with exogenous donor DNA to enable accurate substitutions or insertions [49,50,51,52]. *Deinococcus radiodurans* maintains high HR fidelity even under extreme conditions via the cooperative action of RecA and PprA [53]. Notably, *E. coli* lacks a canonical cNHEJ system, resulting in a pronounced HR bias [49].

In contrast, certain bacteria possess Ku–LigD-mediated NHEJ pathways that generate frequent indels during end-joining, thereby reducing the precision and predictability of repair outcomes. *Pseudomonas aeruginosa* (*P. aeruginosa*) and *Agrobacterium tumefaciens* are examples of bacteria harboring functional NHEJ [54,55]. *Mycobacterium tuberculosis* and *M. smegmatis* possess both HDR and NHEJ but tend to shift toward NHEJ under quiescent or stress conditions, which can yield inconsistent editing results [56]. *Sinorhizobium meliloti* contains two independent NHEJ pathways (Ku2–LigD2 and Ku3/Ku4–LigD4). Under stress, the RpoE2 sigma factor induces their transcription, enhancing efficiency. This mechanism can even integrate foreign DNA into DSBs without homology, contributing to horizontal gene transfer and environmental adaptation [57].

Therefore, sophisticated CRISPR–Cas-based bacterial gene editing requires strategies tailored not only to Cas nuclease efficiency but also to strain-specific DNA repair biases (Table 2). When HR dominates, it is effective to coordinate λ-Red (Exo/Bet/Gam) or RecET (RecE/RecT) recombinase expression with Cas-induced cleavage via inducible expression systems and to introduce anti-recutting (PAM/seed-neutralizing) mutations in donor templates [58,59,60]. Although Cas9 generates blunt DSBs that can be cytotoxic, Cas12a produces staggered 5′-overhang DSBs that may promote HDR entry or recombination preference in certain bacterial contexts [61,62]. Moreover, targeting essential genes in bacteria with deficient repair capacity may lead to lethal DNA damage accumulation [14,63]. These vulnerabilities could be leveraged to design targeted antimicrobial strategies against key genes in repair-deficient bacterial or pathogenic strains.

## 4. Antibiotic Resistance Mechanisms and Horizontal Gene Transfer

**Figure 1:**
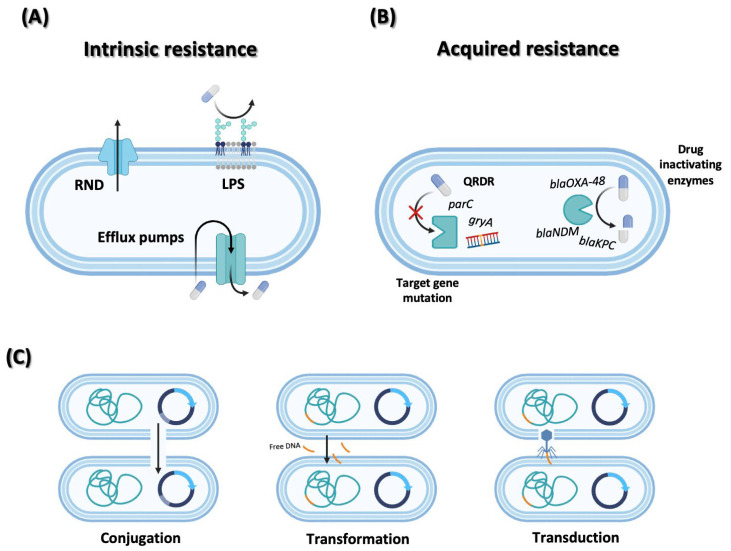
Intrinsic and acquired antibiotic resistance mechanisms and horizontal gene transfer in bacteria. (**A**) Intrinsic resistance mechanisms, including the reduced outer membrane permeability and LPS barrier of Gram-negative bacteria, as well as multidrug efflux systems such as RND family pumps; (**B**) Acquired resistance arising from target-site mutations—exemplified by QRDR mutations in *gyrA* and *parC* that reduce fluoroquinolone binding—and from horizontally acquired carbapenemase genes (*blaKPC*, *blaNDM*, *blaOXA-48*) that inactivate β-lactams; (**C**) Horizontal gene transfer pathways driving the spread of resistance genes: conjugation via plasmid or ICE transfer, transformation by uptake of extracellular DNA, and phage-mediated transduction. Created in BioRender. Yoon, B. (2026) https://app.biorender.com/illustrations/6961b2bf662967415e6cda9c (accessed on 6 December 2025).

The global spread and long-term persistence of antimicrobial resistance result from the complex interplay of evolutionarily conserved bacterial defense strategies. This resistance is not the result of a single mutation but a systemic phenomenon driven by multilayered alterations in cellular structure, metabolism, and genetic adaptation. These sophisticated resistance mechanisms are broadly classified into intrinsic resistance (naturally encoded) and acquired resistance (evolutionarily gained) [64] (Figure 1).

Intrinsic resistance arises from the inherent structural and physiological features of bacteria. For example, the outer membrane and lipopolysaccharide (LPS) barrier of Gram-negative bacteria restrict the penetration of hydrophobic antibiotics, while RND- and MFS-family efflux pumps actively expel intracellular drugs to maintain subtoxic concentrations [64,65,66,67]. In contrast, acquired resistance—directly associated with clinical treatment failure—emerges from target gene mutations or the acquisition of drug-inactivating enzymes. Mutations within the quinolone resistance–determining region (QRDR) of *gyrA* or *parC* reduce fluoroquinolone binding affinity, whereas the acquisition of carbapenemase genes such as *blaKPC*, *blaNDM*, and *blaOXA-48* abolishes β-lactam activity entirely [68,69]. Under antibiotic-selective pressure, these adaptations accumulate rapidly, creating pronounced phenotypic divergence between susceptible and resistant strains within the same lineage.

The principal driver accelerating the dissemination of acquired resistance is horizontal gene transfer, which facilitates the exchange of genetic material across species and genus boundaries. Transformation occurs when naturally competent bacteria uptake and integrate environmental DNA into their chromosomes, as exemplified by the acquisition of *ermB* (macrolide resistance) and *tetM* (tetracycline resistance) genes in *Streptococcus pneumoniae* and *Acinetobacter baumannii* [70,71]. Transduction, mediated by bacteriophages, transfers host-derived DNA fragments between bacteria and occurs widely in environments rich in phage–bacteria interactions, including wastewater, soil, marine habitats, and animal intestines [72,73,74]. This phage-mediated exchange serves as a major evolutionary pathway driving novel resistance gene assemblies within microbial communities.

Among HGT mechanisms, bacterial conjugation is the most clinically significant. Through this process, bacteria exchange conjugative plasmids or integrative conjugative elements (ICEs) via direct cell-to-cell contact, promoting the global dissemination and stabilization of high-risk resistance determinants such as *blaNDM-1*, *blaKPC*, and *mcr-1* [75,76,77]. Especially, integrons enhance multidrug resistance by capturing, rearranging, and expressing diverse gene cassettes. In *P. aeruginosa*, super-integron structures serve as key genetic platforms that accelerate resistance gene accumulation and expression [78,79]. Furthermore, HGT can introduce foreign genes not only during the spread of antibiotic resistance but also during CRISPR-based gene editing processes, thereby disrupting the editing system and reducing overall editing efficiency. This makes it a significant consideration factor in biotechnological interventions.

Resistant bacteria and resistance genes circulate within interconnected ecological networks linking soil, water, livestock, and humans. From a One Health perspective, AMR transcends the boundaries of species and environments, being continually redistributed through recurrent HGT, phage-mediated transfer, and plasmid recombination, thereby fueling global resistance proliferation [80,81]. Therefore, future strategies to mitigate antibiotic resistance should be driven by multidisciplinary collaboration that integrates microbiology, clinical medicine, environmental science, and public health to comprehensively address both resistance mechanisms and transmission dynamics, highlighting the essential role of therapeutic interventions in interrupting the spread of resistance. The importance of therapeutic interventions to block the spread of drug resistance is increasingly emphasized, and in this context, the significance of CRISPR-based interventions enabling precise gene regulation is also gradually gaining prominence.

## 5. Application of the CRISPR-Cas System to Overcome Antimicrobial Resistance

**Figure 2:**
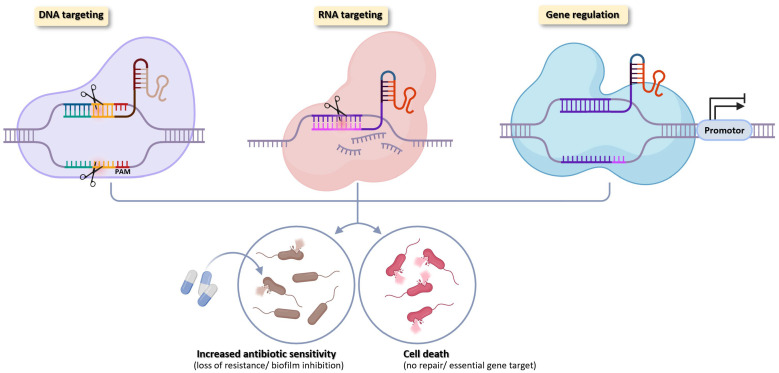
CRISPR-based DNA, RNA, and transcriptional targeting strategies to overcome antibiotic resistance CRISPR–Cas systems act at three levels to counter antibiotic resistance. DNA-targeting nucleases disrupt chromosomal or plasmid loci encoding resistance, virulence, or biofilm functions. RNA-targeting systems degrade specific transcripts to suppress resistance gene expression without genomic alteration. CRISPR-based transcriptional regulation, using dCas-mediated repression or activation, modulates expression of resistance, biofilm, or essential genes. Together, these strategies enable programmable resensitization of bacteria to antibiotics or targeted killing. Created in BioRender. Yoon, B. (2026) https://app.biorender.com/illustrations/6961b27817e0d616fd9e0a5f (accessed on 6 December 2025).

The CRISPR–Cas system is emerging as a next-generation precision tool for reversing antimicrobial resistance in pathogenic bacteria. Unlike conventional antibiotics that exert broad-spectrum selective pressure, CRISPR-based antimicrobials can precisely regulate antibiotic susceptibility and resistance-related genes at the DNA, RNA, or transcriptional level, due to their exceptional programmability and sequence specificity, thereby inducing either bactericidal effects or restoring antibiotic sensitivity [14,82,83,84,85] (Figure 2).

Promising CRISPR targets for restoring or enhancing antibiotic susceptibility are diverse. In addition to canonical resistance genes such as *blaNDM*, *blaKPC*, *mecA*, *vanA*, and *tetM* [85], target candidates include biofilm-associated genes (*icaA*, *gelE*, *csgD*, *mrkA*, *gtfB*) [86]; motility-related genes (*fli*, *mot*, *pil* families) [87,88,89]; and quorum-sensing regulators (*luxS*, *agr* families) [90,91]. These genes play pivotal roles in maintaining antibiotic tolerance, biofilm formation, and virulence; therefore, CRISPR-based regulation of these genes can either directly restore antimicrobial susceptibility or synergistically enhance the activity of co-administered antibiotics (Table 3).

### 5.1. DNA Targeting

Cas9-based CRISPR systems selectively suppress resistant strains by cleaving resistance genes or removing plasmids. For example, CRISPR–Cas9 targeting virulence genes in *Staphylococcus aureus* specifically eliminated pathogenic strains, while plasmid clearance prevented dissemination of resistance among commensal bacteria [14,92].

The pCasCure system, introduced via electroporation into carbapenem-resistant *Enterobacteriaceae* (CRE), efficiently removed *blaNDM*, *blaKPC*, and *blaOXA-48* plasmids with a 94% cure rate, restoring carbapenem susceptibility and enabling effective treatment [93]. Importantly, pCasCure demonstrated that CRISPR–Cas9 can simultaneously target carbapenemase genes and essential plasmid maintenance elements, such as replication and partitioning genes, enabling robust curing of epidemic resistance plasmids across multiple clinically relevant Enterobacteriaceae species. However, when CRISPR–Cas9 was applied to clinical isolates of *Klebsiella michiganensis*, only approximately 63% of transformants exhibited reduced carbapenem resistance, underscoring the complexity of resistance reversal in clinical settings [84]. Detailed genomic and transcriptional analyses revealed that this incomplete resensitization was associated with reduced plasmid copy number and decreased *blaKPC* expression, rather than complete plasmid elimination. Adaptive bacterial responses, including downregulation of the outer membrane porin ompK36 and a frameshift mutation in the efflux pump gene *acrB*, were also identified and likely compensated for the loss of carbapenemase activity. These findings indicate that although CRISPR–Cas9-mediated targeting of a single resistance determinant can substantially weaken resistance, coordinated regulation of porins and efflux systems may limit full phenotypic reversion, thereby emphasizing the need for multiplex or combinatorial CRISPR-based strategies.

Taken together, these studies demonstrate that DNA-targeting CRISPR–Cas9 systems enable highly selective elimination or functional inactivation of resistance determinants at their genetic source, while minimizing collateral damage to non-target bacteria. This sequence-specific DNA targeting offers a fundamental advantage over conventional antibiotics and provides a rational framework for precision antimicrobial strategies aimed at dismantling resistance reservoirs [94].

### 5.2. RNA Targeting

The CRISPR–Cas13 system, which directly targets RNA, is gaining attention as an innovative platform for overcoming bacterial resistance. Cas13a is an RNA-guided nuclease that recognizes and cleaves resistance gene–derived transcripts. When packaged into a bacteriophage capsid (CapsidCas13a), it exhibits potent, sequence-specific bactericidal activity against carbapenem-resistant *E. coli* and methicillin-resistant *S. aureus* (MRSA). Upon target RNA recognition, Cas13a activation triggers collateral RNA cleavage, degrading essential RNAs and leading to bacterial growth inhibition or death [95].

The phagemid-based AB-capsid system further optimized antimicrobial efficacy by increasing phagemid copy numbers and ensuring high packaging efficiency through regulation of *terL–terS–rinA–rinB*. Incorporating CRISPR–Cas13a with crRNAs targeting resistance genes (*mecA*, *aph/aac*, *ermB*, *mphC*, *fusC*, *tetK*), the system selectively eliminated MRSA in a sequence-specific manner via collateral RNA cleavage, while minimizing contamination by wild-type phages [96].

Unlike DNA editing, RNA targeting is reversible and transient, reducing the likelihood of compensatory mutations. Its dual capacity for gene detection and suppression also supports development of “theranostic” platforms for both diagnosis and treatment of resistant infections.

### 5.3. Transcriptional Regulation

CRISPR interference (CRISPRi) employs catalytically inactive dCas proteins to repress gene expression without inducing DNA cleavage. For example, CRISPRi-mediated silencing of *czcR* reduced *mexAB–oprM* efflux pump expression in *P. aeruginosa*, increasing susceptibility to levofloxacin and amikacin [97]. Similarly, repression of *tetA*, *bla*, and *mcr-1* in *E. coli* clinical isolates enhanced susceptibility to meropenem, colistin, and cefotaxime by up to fourfold, confirming the practical potential of CRISPRi-based antibiotic re-sensitization [98].

In multidrug-resistant *Klebsiella pneumoniae* (ST23), an all-in-one CRISPRi system (*pdCas9–gRNA*) targeting *blaNDM-1* and *blaSHV-12* improved susceptibility to meropenem and aztreonam by up to 1000-fold [99]. In this context, this CRISPRi-based strategy achieved potent antibiotic re-sensitization without inducing plasmid loss or double-strand DNA breaks, thereby maintaining native plasmid stability and enabling tunable, inducible suppression of resistance gene expression. This highlights CRISPRi’s value as a precise tool for functional analysis of multidrug-resistance plasmids and for restoring antibiotic sensitivity [100]. Efflux pump-mediated multidrug resistance can also be effectively modulated through CRISPRi-based transcriptional control. An inducible CRISPRi system targeting the AcrAB–TolC efflux pump in *Escherichia coli*, using sgRNAs directed against *acrA*, *acrB*, and *tolC*, demonstrated strong repression of efflux activity. Notably, simultaneous inhibition of *acrB* and *tolC* produced the greatest effect, leading to substantial downregulation of efflux gene expression and resulting in 2- to 16-fold increases in susceptibility to antibiotics such as rifampicin, erythromycin, and tetracycline, along with reduced biofilm formation. These findings highlight CRISPRi-mediated efflux pump regulation as an effective strategy for suppressing efflux-driven resistance and limiting multidrug resistance development in *E. coli*. Conversely, CRISPR activation (CRISPRa) upregulates specific genes by fusing transcriptional activator domains to dCas proteins. Evaluation of key transcriptional regulators involved in resistance—such as SoxS, MarA, and Rob—has enabled the development of CRISPRa systems capable of modulating complex antibiotic resistance networks [101]. Notably, bacterial CRISPRa exhibits strong positional dependence, requiring guide RNAs to be precisely targeted to narrow windows upstream of transcription start sites for effective activation. In addition, CRISPRa can be integrated with CRISPRi and placed under inducible promoters, enabling dynamic, reversible, and simultaneous activation and repression of resistance-associated genes in response to external stimuli.

Overall, CRISPRa/i approaches provide reversible, non-lethal, and double-strand break–free regulation of bacterial genes, minimizing off-target effects and cellular stress. These systems represent promising tools for dissecting and modulating AMR pathways with high precision.

## 6. Delivery Platforms

The successful clinical application of CRISPR–Cas-based target-specific antimicrobial strategies depends not only on precise molecular editing capabilities but also on the ability to deliver them stably and efficiently into bacteria. Bacteria employ multi-layered defense systems—including restriction-modification systems, efflux pumps, biofilms, and anti-phage immunity—to block foreign nucleic acids [85,102,103]. Consequently, developing effective delivery strategies is considered the most significant bottleneck for CRISPR therapeutics. Recent research has explored plasmids, phagemids, nanoparticles, conjugative plasmids, and hybrid platforms, with reported cases not only of resistance gene elimination and antibiotic susceptibility restoration but also of bacterial killing [104,105,106,107,108,109,110,111,112,113,114,115,116,117].

### 6.1. Plasmid

Plasmids represent the earliest CRISPR delivery vectors and played a pivotal role in early proof-of-concept demonstrations. Gomaa et al. demonstrated that the Type I-E CRISPR–Cas system in *E. coli*, when delivered via electroporation, could selectively eliminate target bacterial strains in a sequence-specific manner. They demonstrated that quantitative control of strain ratios within a mixed culture could be achieved by tuning the composition of CRISPR RNAs, highlighting potential applications in the development of “smart antibiotics” and precision modulation of microbial communities [104]. Importantly, this study demonstrated that plasmid-delivered, genome-targeting CRISPR systems can selectively remove closely related bacterial strains in pure and mixed cultures based on sequence-specific spacer design. In mixed-population experiments, modulation of the ratio between targeting and non-targeting CRISPR plasmids enabled titratable control over strain abundance, allowing quantitative adjustment of microbial population structure rather than indiscriminate eradication.

Subsequently, a plasmid-based CRISPR–Cas9 platform was designed to inhibit the dissemination of antimicrobial resistance by blocking HGT. In this system, CRISPR arrays targeting eight distinct resistance genes were incorporated into a plasmid, which reduced resistance gene transfer via transformation in *E. coli* MG1655 and *E. coli* Nissle 1917 by 2–3 log units [105]. Notably, this system conferred protection against the acquisition of resistance genes without eliminating the host bacteria, thereby preserving bacterial viability while selectively removing incoming AMR determinants. Furthermore, the platform was shown to protect a clinically used probiotic strain (*E. coli* Nissle 1917) from horizontal acquisition of resistance plasmids, underscoring the potential of plasmid-based CRISPR delivery for probiotic biocontainment and AMR risk mitigation. This strategy provides a versatile tool for ensuring probiotic strain safety and developing CRISPR-based technologies to curb the spread of resistance.

### 6.2. Conjugative Plasmid

Conjugative plasmids are emerging as powerful delivery vehicles for spreading CRISPR payloads across bacterial populations via HGT. These self-replicating plasmids can transmit genetic cargo through direct cell-to-cell contact, making them ideal for population-level dissemination of CRISPR systems. This approach has been leveraged to suppress the spread of antibiotic resistance and virulence factors by enabling targeted gene removal.

In an *Enterococcus faecalis* model, the donor strain was orally administered to introduce a conjugative CRISPR-Cas system into the mouse gut. Subsequently, through plasmid-mediated conjugation, the system was transferred to recipient strains, enabling the selective removal of *vanA*, *ermB*, and *tetM* resistance genes and significantly reducing the proportion of resistant bacteria within the gut microbiota [106]. Similarly, a conjugative CRISPR–Cas9 platform designed to target *mcr-1*–carrying plasmids in *E. coli* successfully restored polymyxin susceptibility and even induced immunity against re-acquisition of the same plasmid [107]. Furthermore, this system utilized a host-independent conjugative plasmid capable of autonomously disseminating the CRISPR–Cas9 machinery via bacterial conjugation, enabling efficient elimination of *mcr-1*–harboring plasmids across recipient populations. In addition, the engineered CRISPR–Cas9 construct not only cured pre-existing resistance plasmids but also blocked subsequent horizontal transfer by preventing plasmid acquisition through both conjugation and transformation, thereby providing durable protection against the spread of colistin resistance. These studies demonstrate that CRISPR delivery via conjugative plasmids functions analogously to a gene-drive mechanism, enabling the elimination of resistance determinants and the propagation of antibiotic susceptibility throughout bacterial populations.

More recently, a conjugative delivery strategy employing the RNA-targeting CRISPR–Cas13a system has been proposed. Cas13a recognizes and cleaves specific single-stranded RNA sequences guided by its crRNA, leading to the selective killing of pathogens through inhibition of transcription and protein synthesis. Delivery of CRISPR–Cas13a into *Salmonella enterica* serovar Typhimurium via conjugative plasmids effectively suppressed bacterial growth and colony formation in both in vitro and murine infection models, demonstrating pathogen-specific precision without affecting commensal bacteria Bacterial conjugation-mediated delivery of CRISPR–Cas13a to *Salmonella enterica* serovar Typhimurium has been shown to suppress bacterial growth and colony formation in vitro. In vivo, oral administration of a donor *Escherichia coli* strain carrying the conjugative plasmid enabled intestinal delivery of the CRISPR–Cas13a system, resulting in a significant reduction in pathogen colonization in a murine intestinal infection model, while sparing commensal gut bacteria [108].

### 6.3. Phagemid

Phagemids are a platform that utilizes the bacteriophage capsid to package and deliver CRISPR payloads into bacterial cells. They are considered one of the most clinically promising CRISPR delivery strategies due to their high pathogen specificity, efficient gene transfer, and stable packaging properties. Early studies demonstrated that CRISPR–Cas9 phagemids introduced into *Staphylococcus aureus* effectively eliminated resistance genes and restored antibiotic sensitivity [14]. Furthermore, CRISPR–Cas phages have been shown to selectively remove resistance determinants and restore antibiotic susceptibility in clinical isolates of *E. coli* and *Vibrio cholerae* [15].

A CRISPR phage specifically targeting the *blaNDM-1* gene in *Klebsiella pneumoniae* successfully eradicated carbapenem resistance, highlighting its potential for precision control of resistant pathogens [109]. Subsequent work delivered CRISPR–Cas9 to *E. coli* via non-replicative phagemid particles simultaneously targeting *blaCTX-M* group 1 and 9 variants, which significantly reduced the proportion of resistant cells and reversed cephalosporin resistance [110]. By designing multiplex spacers capable of simultaneously silencing multiple AMR variants, this approach demonstrates a feasible strategy for limiting the spread of multidrug resistance.

RNA-targeting CRISPR–Cas13a phagemids have also been developed, showing strong sequence-specific activity and the ability to selectively eliminate resistant bacteria [95]. Moreover, a hybrid phagemid–capsid system achieved precise elimination of *Staphylococcus aureus* (MRSA) with low contamination rates and high packaging efficiency, confirming its potential as a scalable therapeutic delivery vehicle [96].

Most recently, the CRISPR-associated transposase (DART) system was integrated into bacteriophage λ, enabling large-scale gene insertion and targeted disruption within bacterial genomes. This platform achieved over 50% editing efficiency in *E. coli* within mixed microbial communities, offering a highly adaptable tool for in situ genome manipulation and regulation of microbial community composition [111].

### 6.4. Nanoparticle

Nanoparticles have emerged as a promising non-viral delivery platform for CRISPR payloads, offering efficient transport of genetic material or therapeutic agents into diverse bacterial species while minimizing host immune responses. Their high penetration and targeting capabilities make them particularly suitable for bacterial infections. Encapsulation of a Cas13a expression vector within lipid nanoparticles (LNPs) containing the cationic lipid DOTAP enabled effective bacterial transfection and infection control in a clinical *Escherichia* infection model using an intraperitoneal delivery approach. Moreover, co-administration with polymyxin B at non-cytotoxic concentrations further enhanced therapeutic efficacy, demonstrating synergistic antimicrobial activity [112].

Another study utilized biosimilar hybrid vesicles (BCVs) created by fusing Gram-negative bacterial outer membrane vesicles (OMVs) with cationic lipids to deliver CRISPR–Cas9 plasmids in vivo. This delivery method demonstrated significant therapeutic efficacy, including via intratracheal administration in an *A. baumannii* lung infection model and local application in a *P. aeruginosa* wound infection model [113]. Likewise, carbon quantum dot (CQD)-based nanocomplexes (CRISPR-dots) targeting the *papG* adhesion factor of uropathogenic *E. coli* (UPEC) effectively suppressed gene expression, resulting in reduced bacterial adhesion, biofilm formation, and virulence, while markedly improving host survival [114]. Additionally, polymer-derivatized CRISPR nanocomplexes targeting *mecA* in *S.aureus* demonstrated high delivery efficiency and potent target-specific bactericidal activity against MRSA, underscoring the potential of direct CRISPR delivery strategies for resistant pathogens [92].

Nanoparticle-mediated delivery thus represents an advanced and modular alternative that enhances target specificity, protects nucleic acids from degradation, and facilitates biofilm penetration, while minimizing host restriction and unintended dissemination risks associated with phage- or plasmid-based vectors. Moreover, these multifunctional nanocomplexes can co-deliver antibiotics, pathogen-specific antibodies, or antimicrobial peptides, further amplifying synergistic antimicrobial effects [115,116,117]. Despite their promise, key challenges for clinical translation remain—particularly in optimizing formulation stability, minimizing off-target interactions, and establishing long-term biosafety.

## 7. CRISPR-Based Regulation of Antibiotic Susceptibility: Therapeutic Potential and Limitations

CRISPR-based strategies for regulating antibiotic susceptibility present an innovative therapeutic paradigm by precisely targeting resistance genes and virulence factors at the sequence level, thereby overcoming the broad and non-specific limitations of existing antibiotics [20,46,85]. Extensive preclinical evidence now demonstrates that multi-layered CRISPR interventions—encompassing Cas9-mediated DNA cleavage, Cas13-driven RNA degradation, and dCas9-based CRISPRa/i transcriptional regulation—can restore antibiotic susceptibility, eliminate resistance plasmids, and disrupt biofilm integrity [93,94,98,99,112].

Importantly, these approaches have been experimentally validated in several clinically relevant multidrug-resistant (MDR) and extensively drug-resistant (XDR) bacterial pathogens, including carbapenem-resistant *Escherichia coli* and *Klebsiella pneumoniae*, vancomycin-resistant *Enterococcus* spp., and *Salmonella enterica*. In these systems, CRISPR-mediated targeting resulted in resistance gene elimination, transcriptional resensitization, or pathogen-specific growth suppression in both in vitro and in vivo models [84,93,96,97,106,107,108].

Nevertheless, several critical limiting factors currently constrain the clinical translation and long-term robustness of CRISPR-based strategies. The most prominent bottlenecks impeding clinical translation are delivery and specificity. Bacterial restriction–modification systems, efflux pumps, and biofilms significantly hinder the intracellular delivery of nucleic acids [64,67], while in vivo, delivery platforms such as phages, phagemids, conjugative plasmids, and nanoparticles each face inherent limitations related to host range, stability, and immunogenicity [6,7,8]. Expression-based delivery methods (via plasmid or chromosomal integration) also risk the *cas9* gene itself becoming a target of resistance evolution [118]. Although direct delivery of Cas–gRNA ribonucleoprotein (RNP) complexes circumvents this issue, achieving uniform, population-level penetration and sufficiently sustained intracellular activity remains technically challenging, particularly in heterogeneous or biofilm-associated infections. Therefore, optimizing the delivery system based on paper-derived capsids and lipid-polymer nanocomposites can serve as a key step to overcome delivery barriers while maintaining safety [85].

Regarding specificity, off-target cleavage by Cas9 and collateral RNA degradation by Cas13 may disrupt commensal microbiota and perturb host–microbe homeostasis [119,120]. Additionally, mutations within PAM sequences or anti-CRISPR protein expression can accelerate the emergence of CRISPR-resistant subpopulations [85,121]. Crucially, even when virulence factors or antibiotic resistance genes are precisely targeted, it cannot be ensured that resistance development will be completely prevented. Bacteria may adapt through compensatory mutations, activation of alternative resistance pathways, horizontal acquisition of functionally redundant genes, or regulatory network rewiring. Thus, CRISPR-based antibiotic susceptibility control strategies do not eliminate evolutionary pressure; rather, they reconfigure the selective environment in which resistance can arise.

To counter these challenges, the engineering of high-fidelity Cas variants, low- or PAM-free nucleases, and multiplexed gRNA targeting strategies is vital [122]. For expression-based systems, maintaining stable Cas activity may be achieved through chromosomal integration, codon optimization, low-copy plasmid maintenance, or toxin–antitoxin stabilization modules. Introducing spatiotemporal control mechanisms—via light-inducible, ligand-dependent, or split-Cas systems—can further enhance therapeutic precision by restricting CRISPR activation to infection sites or pathogen-specific environments [43,44]. Integration with synthetic biology circuits, such as quorum-sensing or hypoxia-responsive promoters, can confine activity to pathogenic microenvironments, minimizing collateral effects on normal microbiota [123].

Moreover, combination therapies that pair CRISPR systems with antibiotics or antimicrobial peptides show synergistic benefits, enhancing membrane permeability, suppressing compensatory resistance pathways, and reducing side effects by enabling lower drug dosages [115,116,117]. However, CRISPR–antibiotic combination strategies also present important limitations, such as excessive cellular stress responses that may interfere with phage- or nanoparticle-mediated delivery, reduce CRISPR expression efficiency, disrupt symbiotic microbiota, and generate unpredictable resistance trajectories due to complex selective pressures. Consequently, precise optimization of treatment timing, dosage, and target selection is essential to achieve stable and reproducible synergy.

Beyond individual treatments, CRISPR applications extend to population and environmental dimensions. Engineered probiotic strains carrying CRISPR systems can selectively purge resistance plasmids from intestinal reservoirs and suppress HGT by targeting *tra* or *int* operons [124]. To extend these considerations into broader ecological and public health contexts, the key point is that from a One Health perspective, CRISPR technology offers a unique opportunity to interrupt the ecosystem circulation of antibiotic-resistant ‘superbugs’ across humans, animals, aquatic environments and soil ecosystems. By selectively removing resistance determinants from environmental reservoirs, blocking plasmid-mediated spread within livestock microbiomes, and suppressing antibiotic resistance influx via HGT between interconnected ecological habitats, CRISPR-based tools can function as a precision platform for global superbug suppression and cross-ecosystem antibiotic resistance control. Therefore, integrating CRISPR strategies into One Health-oriented surveillance and intervention systems can strengthen sustainable antibiotic stewardship at both clinical and ecological scales.

However, the dissemination of genetically modified organisms introduces additional biosafety and ecological uncertainties, necessitating continuous risk assessment and long-term monitoring. Accordingly, the integration of artificial intelligence (AI) is transforming CRISPR research and development. AI-assisted design platforms enhance guide RNA accuracy, off-target prediction, and experimental success rates. The integration of AI-enabled automation systems is revolutionizing CRISPR workflows by coordinating experimental design, execution, and data analysis within unified digital infrastructures, thereby improving reproducibility and accelerating innovation. Indeed, these systems automate end-to-end workflows—from gRNA design to NGS analysis and phenotype validation—enabling novices to achieve 80–90% efficiency in a single attempt [125,126]. Collectively, these technological and digital advances elevate sequence-targeted antibiotic susceptibility approaches from experimental alternatives to precision treatment platforms that explicitly incorporate evolutionary and ecological contexts, with this digital convergence laying the foundation for next-generation precision gene therapies.

## 8. Conclusions

In conclusion, CRISPR-based strategies for controlling antibiotic susceptibility function as powerful precision therapies that complement existing antibiotics in the short term while possessing innovative potential as independent treatments capable of suppressing antibiotic resistance at the population level in the long term. By directly targeting resistance genes, virulence factors, and mobile genetic elements, CRISPR systems offer unprecedented specificity and adaptability in infection control.

Nevertheless, the successful clinical and ecosystem application of this technology hinges on overcoming key limitations related to delivery efficiency, specificity, evolutionary evasion, and biosafety. Continued advancements in vector optimization, high-precision low-PAM Cas engineering, multi-gRNA design, spatiotemporal regulation, rational combination therapies, and AI-based design automation are expected to accelerate this transition. In parallel, systematic detection and genomic characterization of endogenous CRISPR–Cas systems across diverse bacterial species will be essential to determine when native CRISPR machinery can be harnessed versus when exogenous CRISPR platforms are required. Collectively, these innovations establish the foundation for CRISPR-based antibiotic susceptibility control strategies to set new standards for precision infection management, global superbacteria transmission control, and sustainable antibiotic stewardship within the One Health framework.

## Figures and Tables

**Table 1:** Structural features and applications of CRISPR enzymes.

Type	Enzyme	Target	Key Features	Main Applications	Reference
Cas9 Family	SpCas9	DNA DSB	Widely used; high efficiency; potential off-targets; large size unsuitable for AAV delivery	In vivo gene editing	[31]
	SaCas9	DNA DSB	Compact; AAV-compatible; high specificity and efficiency	In vivo gene editing	[23]
	CjCas9, NmCas9	DNA DSB	Very small; restricted PAMs; lower activity in mammalian cells	Compact genome editing	[24]
	eSpCas9, HF-Cas9, HypaCas9	DNA DSB	Engineered for high fidelity; minimized off-target cleavage	High-precision genome editing	[32,33,34]
	xCas9, SpCas9-NG, SpRY	DNA DSB	Expanded PAM compatibility (NG, NRN, NYN); variable efficiency	Broad-range genome targeting	[30,31]
	dCas9	Binding only	Catalytically inactive; DNA binding without cleavage	CRISPRi/a, gene visualization, regulation	[38]
Cas12 Family	nCas9 (D10A, H840A)	Single-strand nicking	Generates single-strand nicks; enhances HDR precision; reduces indels	Precision repair and hybrid editing	[39]
	Cas12a (Cpf1)	DNA DSB (5′ overhang)	Recognizes 5′ TTTV PAM; single crRNA; comparatively low off-targets; multiplex editing possible	Genome editing, diagnostics	[16,17]
	Cas12j (CasΦ)	DNA	Ultra-small Cas nuclease; potential activity in mammalian systems	Compact in vivo editing	[25]
	Cas12f1 (CasX)	DNA	Small, programmable, minimal off-targets	Compact delivery, in vivo editing potential	[26]
	Cas12f2 (Cas14)	ssDNA	ssDNA-specific; strong collateral cleavage; highly specific	Molecular diagnostics, multiplex detection	[27]
Functionally Expanded Cas Enzymes	Cas13a/b/d	RNA	RNA-targeting CRISPR; no PAM required; collateral RNase activity	RNA editing, SHERLOCK diagnostics	[16,17]
	Base editor	Base substitution	DSB-free nucleotide conversion (A→G, C→T); Cas–deaminase fusion	Precise base editing	[40]
	Prime editor	Base substitution/small indel correction	Cas9 nickase + reverse transcriptase; DSB-free editing	Precision genome correction	[41]
	CRISPRa	Activation control	dCas9-VP64 fusion; transcriptional upregulation	Gene activation	[38]
	CRISPRi	Repression control	dCas9-KRAB fusion; transcriptional repression	Gene silencing	[38]
	paCas9/split-Cas9/ligand-inducible variants	Conditional activation	Spatial/temporal control; ligand- or light-induced activation; enhanced safety	Controlled in vivo regulation	[42,43]

Abbreviations: AAV, adeno-associated virus; Cas, CRISPR-associated protein; crRNA, CRISPR RNA; CRISPRa, CRISPR activation; CRISPRi, CRISPR interference (transcriptional repression); DSB, double-strand break; HDR, homology-directed repair; indel, insertion or deletion mutation; PAM, protospacer adjacent motif; ssDNA, single-stranded DNA; TTTV, thymine-rich PAM motif (T/T/T/V, where V = A/C/G); VP64, tetramer of the VP16 transcriptional activation domain. Notes: Activity and PAM specificity may vary across species and experimental contexts. Base and Prime Editors and CRISPRa/i systems represent engineered functional expansions based on Cas backbones rather than distinct natural effectors.

**Table 2:** Representative DNA repair pathways influencing CRISPR–Cas-mediated genome editing outcomes in bacteria.

Repair Pathway	Core Components	Mechanism	Representative Species	Impact on CRISPR Editing	References
Homologous Recombination (HR)	RecA, RecBCD (or AddAB/AdnAB), RecFOR	Template-dependent repair using homologous DNA; precise DSB correction; supports donor DNA integration.	*E. coli*, *H. pylori*, *H. influenzae*, *L. lactis*, *D. radiodurans*	Facilitates precise substitutions or insertions via HDR; enables high-fidelity CRISPR editing.	[45,47,49,50,51,52,53]
Non-Homologous End Joining (NHEJ)	Ku, LigD	Template-independent ligation of DSB ends; error-prone with frequent indels.	*P. aeruginosa*, *A. tumefaciens*, *M. tuberculosis*, *M. smegmatis*, *S. meliloti*	Generates indels; lowers precision; may dominate under stress or stationary-phase conditions.	[54,55,56,57]
Alternative End Joining (A-EJ/MMEJ)	RecA-independent; PolA or LigC; short microhomologies (2–25 bp)	Aligns short microhomologous sequences near break sites; error-prone; backup when HR/NHEJ are inactive.	*B. subtilis*, *Deinococcus* spp., *Mycobacterium* spp.	Produces small deletions/rearrangements; aids survival in repair-deficient strains.	[45,48]
No/Deficient Repair Capacity	Lacking key HR/NHEJ components	DSBs remain unrepaired, causing lethality or genome instability.	Repair-deficient mutants, certain *E. coli* strains	Causes hypersensitivity to Cas-induced DSBs; exploitable for antimicrobial targeting.	[14,63]

**Table 3:** Representative target genes involved in the restoration or enhancement of antibiotic susceptibility.

Category	Representative Genes	Function/Role	Representative Species
Antibiotic resistance–related genes	*blaNDM*, *blaKPC*	Encode carbapenemase enzymes; hydrolyze β-lactam antibiotics including carbapenems	Carbapenem-resistant *Enterobacteriaceae* (CRE)
	*mecA*	Produces penicillin-binding protein 2a (PBP2a) protein; prevents β-lactam binding, leading to methicillin resistance	Methicillin-resistant *Staphylococcus aureus* (MRSA)
	*vanA*	Modifies D-Ala–D-Lac terminus of peptidoglycan, reducing vancomycin binding	Vancomycin-resistant *Enterococcus* (VRE)
	*tetM*	Encodes ribosomal protection protein conferring resistance to tetracyclines binding to the 30S subunit	*Enterococcus faecalis*, *E. coli*
Biofilm- and virulence-associated genes	*icaA*	Synthesizes polysaccharide intercellular adhesin (PIA); promotes intercellular adhesion and biofilm initiation	*Staphylococcus epidermidis*, *S. aureus*
	*gelE*	Encodes gelatinase contributing to biofilm maturation and virulence	*Enterococcus faecalis*
	*csgD*	Transcriptional regulator controlling curli fiber and cellulose biosynthesis; promotes cell adhesion and biofilm development	*E. coli*, *Salmonella* spp.
	*mrkA*	Encodes the major pilin subunit of type 3 fimbriae; mediates adhesion to abiotic surfaces and biofilm formation	*Klebsiella pneumoniae*
	*gtfB*	Synthesizes glucosyltransferase enzymes for extracellular polysaccharide matrix formation	*Streptococcus mutans*
Motility- and adhesion-related genes	*fli* *C*	Encodes flagellin and associated proteins for swimming/swarming motility	*Salmonella enterica*, *Pseudomonas aeruginosa*
	*motA*, *motB*	Forms flagellar stator complex; essential for bacterial motility and energy coupling	*E. coli*, *Salmonella* spp.
	*pilA*, *pilB*, *pilT*	Type IV pili biogenesis; mediates twitching motility and surface attachment	*P. aeruginosa*, *Neisseria* spp.
Quorum-sensing regulators	*luxS*	Regulates AI-2-mediated quorum sensing; coordinates biofilm formation and virulence gene expression	*E. coli*, *Streptococcus mutans*, *Vibrio harveyi*
	*agrA*, *agrB*, *agrC*, *agrD*	Accessory gene regulator (Agr) quorum-sensing system controlling toxin expression and biofilm dispersal	*Staphylococcus aureus*, *Enterococcus faecalis*

## Data Availability

No new data were created or analyzed in this study. Data sharing is not applicable to this study.

## Abbreviations

The following abbreviations are used in this manuscript:

AMR	Antimicrobial resistance
AAV	Adeno-associated virus
A-EJ	Alternative end joining
AI	Artificial intelligence
AGR	Accessory gene regulator
BCV	Biosimilar hybrid vesicle
Cas	CRISPR-associated protein
CQD	Carbon quantum dot
CRISPR	Clustered regularly interspaced short palindromic repeats
CRISPRa	CRISPR activation
CRISPRi	CRISPR interference (transcriptional repression)
CRE	Carbapenem-resistant Enterobacteriaceae
crRNA	CRISPR RNA
DSB	Double-strand break
HGT	Horizontal gene transfer
HDR	Homology-directed repair
HR	Homologous recombination
ICE	Integrative conjugative element
LNP	Lipid nanoparticle
LPS	Lipopolysaccharide
MMEJ	Microhomology-mediated end joining
MRSA	Methicillin-resistant *Staphylococcus aureus*
NHEJ	Non-homologous end joining
OMV	Outer membrane vesicle
PAM	Protospacer adjacent motif
PACE	Phage-assisted continuous evolution
PIA	Polysaccharide intercellular adhesin
PMB	Polymyxin B
QRDR	Quinolone resistance–determining region
RNP	Ribonucleoprotein
SHV	Sulfhydryl variable β-lactamase
TTTV	Thymine-rich PAM motif (T/T/T/V, where V = A/C/G)
UPEC	Uropathogenic *Escherichia coli*
VRE	Vancomycin-resistant *Enterococcus*
